# Metabolomic signature of pediatric diabetic ketoacidosis: key metabolites, pathways, and panels linked to clinical variables

**DOI:** 10.1186/s10020-024-01046-9

**Published:** 2024-12-20

**Authors:** Paolo Spagnolo, David Tweddell, Enis Cela, Mark Daley, Cheril Clarson, C. Anthony Rupar, Saverio Stranges, Michael Bravo, Gediminas Cepinskas, Douglas D. Fraser

**Affiliations:** 1 Medicine, Campus Bio-Medico University of Rome, Via Alvaro del Portillo 21, 00128 Rome, Italy; 2 Computer Science, Western University, London, ON N6A 3K7 Canada; 3 Physiology & Pharmacology, Western University, London, ON N6A 3K7 Canada; 4 Epidemiology and Biostatistics, Western University, London, ON N6G 2M1 Canada; 5 Pediatrics, Western University, London, ON N6A 3K7 Canada; 6 Biochemistry, Western University, London, ON N6A 3K7 Canada; 7 Family Medicine, Western University, London, ON N6G 2M1 Canada; 8 Clinical Medicine and Surgery, University of Naples Federico II, Naples, 80131 Italy; 9 Emergency Department, Hospital for Sick Children, Toronto, ON M5G 1X8 Canada; 10 Medical Biophysics, Western University, London, ON N6A 3K7 Canada; 11 Anatomy and Cell Biology, Western University, London, ON N6A 3K7 Canada; 12 London Health Sciences Centre Research Institute, London, ON N6C 2R5 Canada; 13 Clinical Neurological Sciences, Western University, London, ON N6A 3K7 Canada; 14 Child Health Research Institute, London, ON N6C 4V3 Canada; 15 A5-132, Victoria Research Laboratories, London Health Sciences Centre, Victoria Campus, 800 Commissioners Road E, London, ON N6A 5W9 Canada; 16 Medicine, Western University, London, ON, N6A 3K7, Canada

**Keywords:** Pediatric, Diabetic ketoacidosis, Metabolomics, Metabolites, Pathways, Panels

## Abstract

**Background:**

Diabetic ketoacidosis (DKA) is a serious complication of type 1 diabetes (T1D), arising from relative insulin deficiency and leading to hyperglycemia, ketonemia, and metabolic acidosis. Early detection and treatment are essential to prevent severe outcomes. This pediatric case–control study utilized plasma metabolomics to explore metabolic alterations associated with DKA and to identify predictive metabolite patterns.

**Methods:**

We examined 34 T1D participants, including 17 patients admitted with severe DKA and 17 age- and sex-matched individuals in insulin-controlled states. A total of 215 plasma metabolites were analyzed using proton nuclear magnetic resonance and direct-injection liquid chromatography/mass spectrometry. Multivariate statistical methods, machine learning techniques, and bioinformatics were employed for data analysis.

**Results:**

After adjusting for multiple comparisons, 65 metabolites were found to differ significantly between the groups (28 increased and 37 decreased). Metabolomics profiling demonstrated 100% accuracy in differentiating severe DKA from insulin-controlled states. Random forest analysis indicated that classification accuracy was primarily influenced by changes in ketone bodies, acylcarnitines, and phosphatidylcholines. Additionally, groups of metabolites (ranging in number from 8 to 18) correlated with key clinical and biochemical variables, including pH, bicarbonate, glucose, HbA1c, and Glasgow Coma Scale scores.

**Conclusions:**

These findings underscore significant metabolic disturbances in severe DKA and their associations with critical clinical indicators. Future investigations should explore if metabolic alterations in severe DKA can identify patients at increased risk of complications and/or guide future therapeutic interventions.

**Supplementary Information:**

The online version contains supplementary material available at 10.1186/s10020-024-01046-9.

## Introduction

Type 1 diabetes (T1D) is a global health issue, primarily affecting children and young adults, with incidence rates doubling every decade (Mobasseri et al. 2020). The key pathology is insulin deficiency due to immune-mediated destruction of pancreatic β cells, leading to hyperglycemia (Katsarou et al. 2017). Common symptoms at presentation include weight loss, polyuria, and polydipsia (Nigrovic et al. 2023).

Diabetic ketoacidosis (DKA) is a common complication of T1D, occurring when insulin deficiency is severe or counter-regulatory hormones rise during stress (Segerer et al. 2021; Vicinanza et al. 2019). DKA is diagnosed by hyperglycemia, ketonemia, and metabolic acidosis (Dhatariya et al. 2020; Calimag et al. 2023). If untreated, hyperosmolality, acidosis, and a catabolic state can lead to lethargy, coma, cardiovascular collapse, and death (Siqueira 2011; Bialo et al. 2015). Treatment involves intravenous fluids and insulin (Castellanos et al. 2020; Jayashree et al. 2019), but children and adolescents are particularly vulnerable to complications, notably life-threatening cerebral edema (Segerer et al. 2021; Wolfsdorf et al. 2006).

Metabolomics refers to a set of methodologies for investigating a large spectrum of endogenous metabolites in human fluids and may provide one of the clearest pictures of human phenotype and medical condition. Commonly used metabolomic analytical techniques include nuclear magnetic resonance (NMR) and mass spectrometry (MS). Potential advantages of metabolomics include biomarker discovery, as well as providing a greater understanding of disease pathogenesis and toxicity (Beckonert et al. 2007; Bingol et al. 2015; Markley et al. 2017).

While the metabolic pathophysiology of DKA has been studied, comprehensive pathway analysis is lacking. In adult patients, metabolomics profiling has been used to delineate several altered metabolic pathways in T1D and type 2 diabetes, while a limited number of metabolites have been investigated in a small number of adult DKA patients (Jahoor et al. 2021; Jin and Ma 2021).

In this case–control study, we hypothesized that diabetic ketoacidosis (DKA) would exhibit a distinct metabolite profile compared to insulin-controlled type 1 diabetes (T1D) participants, matched by age and sex. Furthermore, we aimed to identify specific metabolites that are associated with key clinical correlates of DKA and its metabolic consequences, including pH, bicarbonate levels, glucose, HbA1c, and Glasgow Coma Scale (GCS). Our aims were: (1) to measure plasma metabolites with two complementary techniques, direct-injection liquid chromatography/mass spectroscopy (DI-LC/MS/MS) and proton nuclear magnetic resonance (^1^H NMR); (2) to compare metabolomic profiles; and (3) to determine specific metabolite patterns associated with DKA clinical characteristics.

## Methods

### Study design and participants

Patients with T1D were recruited from the Children’s Hospital, London Health Sciences Centre (London, Ontario, Canada). DKA was diagnosed based on hyperglycemia (blood glucose > 11 mmol/L), bicarbonate < 15 mmol/L, and ketonuria, and classified as mild (venous pH < 7.3), moderate (pH < 7.2), or severe (pH < 7.1). We enrolled only patients with severe DKA admitted to the pediatric critical care unit (PCCU) over a two-year period. Insulin-controlled T1D patients, with no history of DKA for at least 3 months, were enrolled from an outpatient clinic. A convenience sampling method was used, as accurate sample size calculations are not feasible in large-scale metabolomic studies where effect size and variance are unknown.

### Blood collection and processing

Blood samples intended for both proteomic analyses and routine DKA laboratory testing were collected at PCCU admission prior to insulin administration. In all cases, normal saline was being administered. Samples were drawn into citrate-containing tubes (Vacutainers; BD Biosciences, Mississauga, Canada) by certified nursing personnel, kept on ice, and promptly transported to the Translational Research Centre facility for processing according to standard operating procedures (www.translationalresearch.ca; London, Canada). The blood was centrifuged at 1,500 x *g* for 15 min at 4 °C, and the upper plasma layer was aliquoted into 250 µL portions. The buffy coat was then removed and aliquoted. Both plasma and buffy coat aliquots were immediately frozen at − 80 °C for later use. For experiments, plasma was thawed and kept briefly on ice, with precautions taken to avoid freeze–thaw cycles.

### DI-LC/MS/MS

A targeted quantitative metabolomics approach was applied to analyze the plasma samples using a combination of direct injection mass spectrometry (AbsoluteIDQ^™^ Kit) with a reverse-phase LC/MS/MS Kit (BIOCRATES Life Sciences AG, Austria). The method combines the derivatization and extraction of analytes, and selective mass-spectrometric detection using multiple reaction monitoring pairs (standards are integrated in the Kit plate filter for metabolite quantification). Briefly, plasma samples were thawed on ice and then vortexed and centrifuged at 13,000 × *g*. Each plasma sample (10 µL) was loaded onto the center of the filter on the upper 96-well kit plate and dried in a stream of nitrogen. Subsequently, 20 µL of a 5% solution of phenyl-isothiocyanate was added for derivatization. After incubation, the filter spots were dried again using an evaporator. Extraction of the metabolites was then achieved by adding 300 µL methanol containing 5 mM ammonium acetate. The extracts were obtained by centrifugation into the lower 96-deep well plate, followed by a dilution step with kit MS running solvent. Mass spectrometric analysis was performed on an API4000 Qtrap^®^ tandem mass spectrometry instrument (Applied Biosystems/MDS Analytical Technologies, Foster City, CA) equipped with a solvent delivery system. The samples were delivered to the mass spectrometer by LC followed by a DI. The Biocrates MetIQ software was used to control the entire assay workflow, from sample registration to automated calculation of metabolite concentrations. A targeted profiling scheme was used to quantitatively screen for known small molecule metabolites using multiple reaction monitoring, neutral loss and precursor ion scans. A total of 178 compounds were analyzed with DI-LC/MS/MS, categorized as follows: acylcarnitines (40), amino acids (20), biogenic amines (12), glycerophospholipids (90), sphingolipids (15), and sugars (1). Notably, many of the lipids and the single sugar (Hexose) represented groups of molecules rather than individual species.

### ^1^H NMR

Plasma samples were deproteinized using ultra-filtration (Psychogios et al. 2011). Prior to filtration, 3 KDa cut-off centrifugal filter units (Amicon Microcon YM-3) were rinsed five times each with 0.5 mL of H2O and centrifuged (10,000 rpm for 10 min) to remove residual glycerol bound to the filter membranes. Aliquots of each plasma sample were then transferred into the centrifuge filter devices and centrifuged (10,000 rpm for 20 min) to remove macromolecules (primarily protein and lipoproteins) from the sample. The filtrates were collected, and the volumes were recorded. The volume of the sample was adjusted with the addition of 50 mM NaH_2_PO_4_ buffer (pH 7.0) until the total volume of the sample was 600 µL. Any sample that had to have buffer added to bring the solution volume to 600 µL, was annotated with the dilution factor and metabolite concentrations were corrected in the subsequent analysis. Subsequently, 70 µL of D_2_O and 30 µL of a standard buffer solution (11.7 mM DSS [disodium 2, 2-dimethyl-2-silcepentane-5- sulphonate], 730 mM imidazole, and 0.47% NaN_3_ in H2O) was added to the sample. The plasma sample (700 µL) was then transferred to a standard NMR tube for subsequent spectral analysis. All 1H-NMR spectra were collected on a 500 MHz Inova (Varian Inc. Palo Alto, CA) spectrometer equipped with a 5 mm HCN Z-gradient pulsed-field gradient room temperature probe. Proton NMR spectra were acquired at 25 °C using the first transient of the NOESY-pre-saturation pulse sequence, chosen for its high degree of quantitative accuracy (Saude et al. 2006). All free induction decays were zero-filled to 64 K data points and subjected to a line broadening of 0.5 Hz. The singlet produced by the DSS methyl groups was used as an internal standard for chemical shift referencing (set to 0 ppm) and for quantification, all 1H-NMR spectra were processed and analyzed using the Chenomx NMR Suite Professional Software package version 7.1 (Chenomx Inc, Edmonton, AB). The Chenomx NMR Suite software allows for qualitative and quantitative analysis of an NMR spectrum by manually fitting spectral signatures from an internal database to the spectrum. Specifically, the spectral fitting for metabolites was performed using the standard Chenomx 500 MHz metabolite library. Typically, 90% of visible peaks were assigned to a compound and more than 90% of the spectral area could be routinely fit using the Chenomx spectral analysis software. Most of the visible peaks are annotated with a compound name. It has been previously shown that this fitting procedure provides an absolute concentration accuracy of 90% or better. Each spectrum was processed and analyzed by at least two NMR spectroscopists to minimize compound misidentification and incorrect quantification. We used sample spiking to confirm the identities of assigned compounds. The NMR data set identified 37 unique metabolites that were predominantly amino acids.

### Statistical analysis

Duplicates were first removed, with DI-LC/MS/MS values preferred over those determined with ^1^H NMR. Normalized data were then used for statistical hypothesis testing to identify metabolites that are significantly different between sample groups. Statistical comparisons were conducted using empirical Bayes moderated t-tests, implemented through the limma R package. P-values were adjusted for multiple testing to control the false discovery rate. For each comparison (e.g., DKA vs. CON), a positive log2(fold change) indicates up-regulation in DKA relative to CON, while a negative log2(fold change) indicates down-regulation.

### Metabolite feature importance in classification

Raw metabolite data from 34 participants were processed to identify key features for classification. A random forest model comprising 20,000 decision trees (maximum depth of 3 leaves) was trained for this classification task. The Boruta feature selection method was subsequently applied to the trained classifier (Kursa and Rudnicki, 2010), resulting in the identification of 54 features deemed significant for classification. A second random forest model (20,000 estimators, maximum depth of 3) was then trained using only these 54 selected features to assess the relative importance of each metabolite.

### Clinical features-metabolite associations

A sparse linear regression was conducted using the L1 norm as a regularizer (Lasso regression with alpha = 0.01) to analyze the relationship between the 20 most important metabolites and each clinical observable. Prior to the Lasso regression analysis, each metabolite was standardized to have a mean of zero and unit variance. The residual sum of squares (RSS) was calculated to evaluate the model's fit to the data. All analyses and visualizations were performed using Python 3.11.8, along with the pandas 2.2, numpy 1.26.4, and mne-connectivity 0.7.0 libraries.

### Pathway analyses

To identify enriched pathways, we utilized the KEGG Homo sapiens pathway library. Pathway enrichment was assessed using the Globaltest statistical technique, which allowed us to quantify the number of compounds associated with each pathway and identify significant hits from the uploaded data. We conducted a comprehensive evaluation of significance by examining raw and adjusted p-values, false discovery rates (FDR), and pathway impact based on topological analysis. Given that our primary objective was to pinpoint the most relevant pathways, the rank of each pathway was prioritized over absolute p-values.

## Results

### Patient variables

**Fig. 1:**
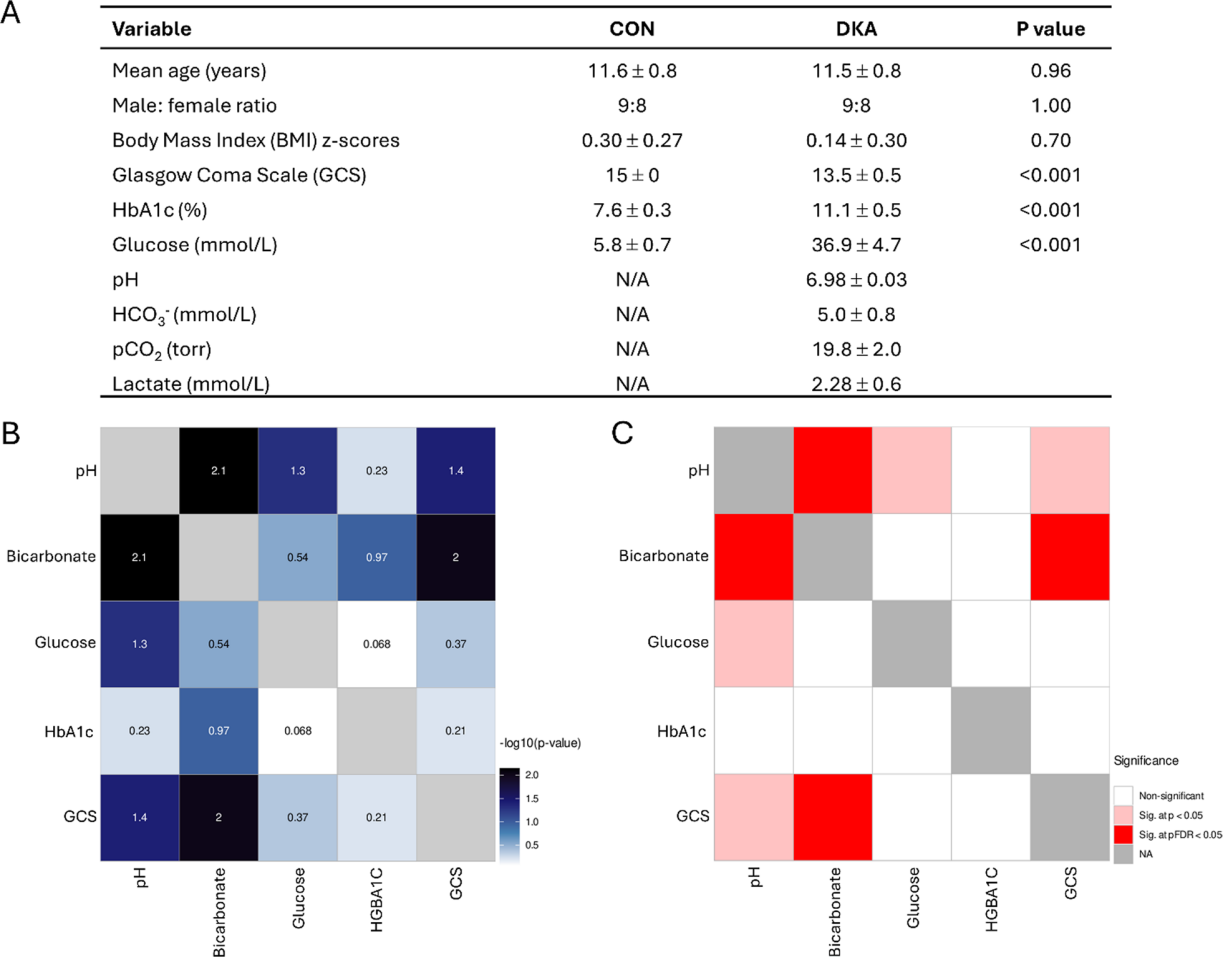
Clinical and biochemical data for DKA patients supplemented with heatmaps showing the strength of association between covariates. **A** T1D diagnosis was based on clinical and biochemical criteria. Type 1 diabetes-related autoantibodies were only measured if the diagnosis was equivocal due to the presence of clinical and demographic risk factors for type 2 diabetes (n = 3; T1D was confirmed by positive islet-cell antibodies and antibodies to glutamic acid decarboxylase. Glasgow Coma Scale (GCS) was determined on admission to the pediatric critical care unit. *CON* Insulin-controlled patients, *DKA* acute Diabetic Ketoacidosis (n = 17/group). BMI and BMI *z*-scores (kg/m2) were calculated from the U.S. Centers for Disease Control and Prevention reference data (n = 14–17/group). Data presented as mean ± SEM. N/A; blood not tested due to lack of clinical indication. **B** Continuous heatmap showing associations between all factors in the study. *P-values* are written as -log10(p-value) and depicted as continuous gradients with darker blue blocks indicating stronger associations. The significant threshold was set at* P* < 0.05 and the *P*-values were adjusted for multiple testing using Benjamini–Hochberg correction. **C** Discrete heatmap showing associations between all factors in the study. The strength of the associations is defined by the color of the blocks as non-significant (white), significant at *p-value* < *0.05* (pink), significant at FDR < 0.05 (red)

The demographic and laboratory values for all study T1D patients are presented in Fig. 1A. The 34 DKA and CON patients were age- and sex-matched, and the body mass index z scores were similar between groups and consistent with T1D. Patients with DKA had significantly lower GCS (GCS 15, n = 8; GCS 14, n = 4; GCS 13, n = 1; GCS 12, n = 1; GCS 10, n = 2; GCS 8, n = 1), suggesting DKA-induced mild brain dysfunction (p < 0.001). Higher HbA1c values were measured in DKA patients as opposed to CON patients (p < 0.001), indicating elevated blood glucose over the previous 2–3 months (Eyth and Naik 2023). DKA patients all had elevated blood glucose, elevated blood ketones and metabolic acidosis on blood gas measurements (p < 0.001). The duration of T1D was 4.1 ± 1.3 years (range 6 months to 10 years; HbA1c 7.6 ± 0.3) for the insulin-controlled participants. Of the DKA patients, 9 were participants with known T1D (duration 3.6 ± 1.7, range 1 to 7 years; HbA1c 9.9 ± 1.6) and 8 participants were newly diagnosed T1D (HbA1c 12.6 ± 1.7). The Hb1Ac measurements from DKA patients that were known T1D versus new onset T1D were significantly different (P = 0.010).

### Metanalyses

Heatmaps illustrate the strength of association between biochemical and clinical covariates in the samples. In Fig. 1B, associations are represented as − log10(p-value), with darker blue blocks indicating stronger associations. Conversely, Fig. 1C uses color coding to denote significance levels: non-significant associations are shown in white, significant at p < 0.05 in pink, and significant at FDR < 0.05 in red. The significance threshold was set at p < 0.05, with p-values adjusted for multiple comparisons using the Benjamini–Hochberg correction. Both heatmaps reveal similar patterns, with two notable correlations: between bicarbonate and pH, and between bicarbonate and GCS scores.

**Fig. 2:**
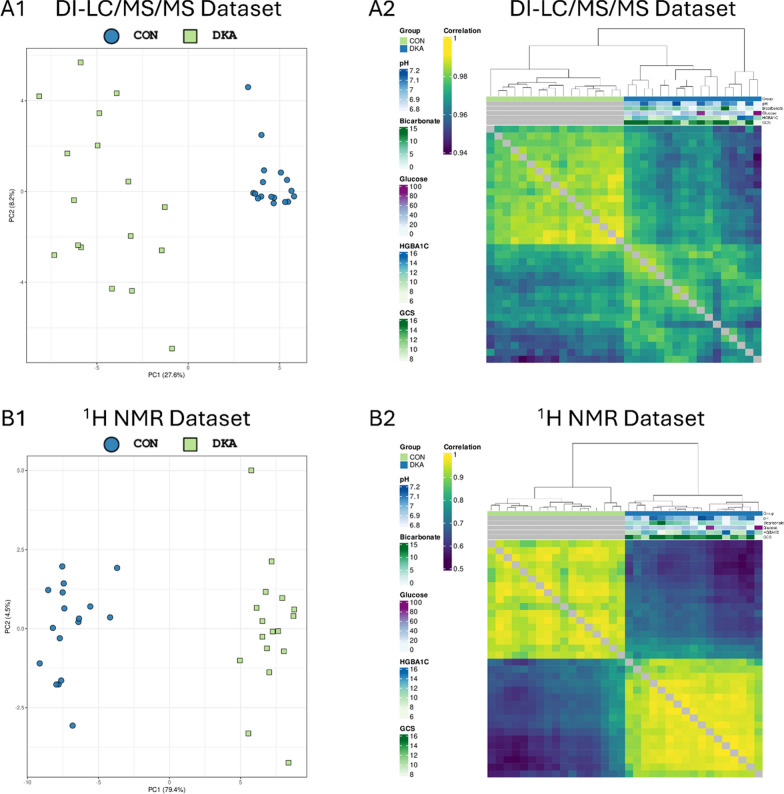
Quality control and exploratory data analysis showing associations, potential grouping, and degree of correlation between samples. **A1** A scatterplot for the first two principal components from the Imputed MS data dataset. Colors represent different factors in the dataset. Samples are expected to cluster according to one or more experimental factors, which might reveal underlying patterns or groupings. **A2** A heatmap showing the between-sample pairwise Pearson correlation of the Imputed MS data. Individual samples are shown along both the X and Y axes, with the degree of correlation indicated by the colors (yellow: higher correlation, purple: lower correlation). Clustering (Euclidean distance) is shown by the dendrograms above and to the left of the image, together with relevant annotation for each sample. **B1** A scatterplot for the first two principal components from the Supplied NMR data dataset. Colors represent different factors in the dataset. Samples are expected to cluster according to one or more experimental factors, which might reveal underlying patterns or groupings. **B2** A heatmap showing the between-sample pairwise Pearson correlation of the Supplied NMR data. Individual samples are shown along both the X and Y axes, with the degree of correlation indicated by the colors (yellow: higher correlation, purple: lower correlation). Clustering (Euclidean distance) is shown by the dendrograms above and to the left of the image, together with relevant annotation for each sample

Figure 2 displays the results of the normalized quality control processes for both MS and NMR data. Principal component analysis (PCA) revealed a clear separation between the cohorts (n = 17/cohort). The scatterplot shows that patients with insulin-controlled diabetes clustered tightly, whereas those with DKA exhibited a more heterogeneous distribution, distinctly separated from the control group. This differentiation highlights significant metabolic differences between the groups and underscores the considerable impact of DKA on the data.

### Metabolites expression and profiling

**Fig. 3:**
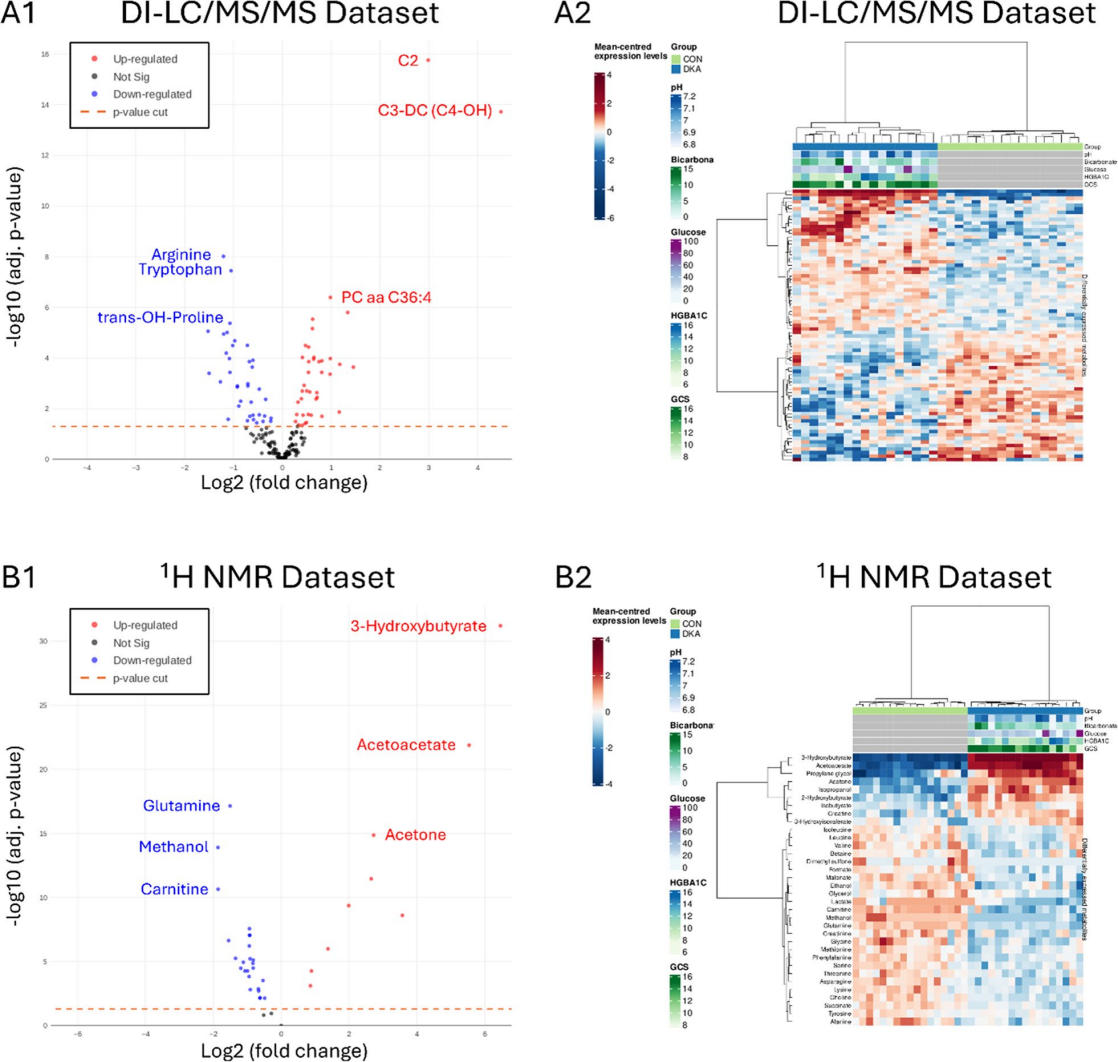
Association tests between DKA patients and controls shown graphically through MS and NMR. **A1** A volcano plot, derived from MS data, showing significance (as -log_10_ transformed p-values) against magnitude (log_2_(fold change)). Metabolites identified as having different levels between samples are represented as red (upregulated) or blue (downregulated) dots, the ones listed by name—arginine, tryptophan, and trans-OH-Proline were downregulated; C2, C3-DC (C4-OH), and PC aa C36:4 were upregulated—were the most significantly altered. To improve performance when there are tens or hundreds of thousands of metabolites the non-significant metabolites (black) displayed are a representative subsample of the full dataset. The horizontal orange line represents the applied p-value threshold. **A2** Heatmap, derived from MS data, shows metabolite intensity per sample relative to the average level across all samples. Individual metabolites are shown on the Y axis while samples are shown along the X axis. Red and blue cells correspond to higher and lower metabolomics levels, respectively. A maximum of 1000 features and 1000 samples are shown (selected at random when the number exceeds these limits). **B1** A volcano plot, derived from NMR data, showing significance (as -log_10_ transformed p-values) against magnitude (log_2_(fold change)). Metabolites identified as having different levels between samples are represented as red (upregulated) or blue (downregulated) dots, the ones listed by name—glutamine, methanol, and carnitine were downregulated; 3-Hydroxyburate, Acetoacetate, and Acetone were upregulated —were the most significantly altered. To improve performance when there are tens or hundreds of thousands of metabolites the non-significant metabolites (black) displayed are a representative subsample of the full dataset. The horizontal orange line represents the applied p-value threshold. **B2** Heatmap, derived from NMR data, showing metabolite intensity per sample relative to the average level across all samples. Individual metabolites are shown on the Y axis while samples are shown along the X axis. Red and blue cells correspond to higher and lower metabolomics levels, respectively. A maximum of 1000 features and 1000 samples are shown (selected at random when the number exceeds these limits)

In plasma, 178 metabolites were identified using DI/LC–MS/MS, while 37 metabolites were identified using NMR. Among these, 34 metabolites identified by NMR and 77 identified by MS were found to be statistically significant with an adjusted p-value < 0.05, some of which exhibited substantial fold changes (Fig. 3). Of the 77 metabolites identified by MS, 37 were downregulated and 40 were upregulated. The most notably upregulated metabolites included tryptophan, arginine, and trans-OH-proline, whereas C2, C3-DC (C4-OH), PC, and PC aa C36:4 was significantly downregulated. Similarly, NMR analysis revealed that 9 out of 34 metabolites were upregulated and 25 were downregulated. The most significantly altered NMR metabolites included the downregulated glutamine, methanol, and carnitine, as well as the upregulated 3-hydroxybutyrate, acetoacetate, and acetone. Supplementary Table 1 presents the combined list of metabolites, both DC/LC–MS/MS and NMR, with their respective adjusted p-values resulting in a total of 65 metabolites that differed significantly between groups (28 increased, 37 decreased).

Feature importance was assessed using random forests, resulting in a ranked list of metabolites contributing to the variance between DKA and CON patients (Supplementary Table 2). A notable finding was the high variability in feature importance across the metabolite dataset. Detailed analysis revealed that key DKA metabolites, such as ketones, were highly ranked, with over half of the top 54 metabolites categorized into just two classes: 19 acylcarnitines (C) and 16 phosphatidylcholines (PC and lysoPC).

### Clinical-metabolite associations

**Fig. 4:**
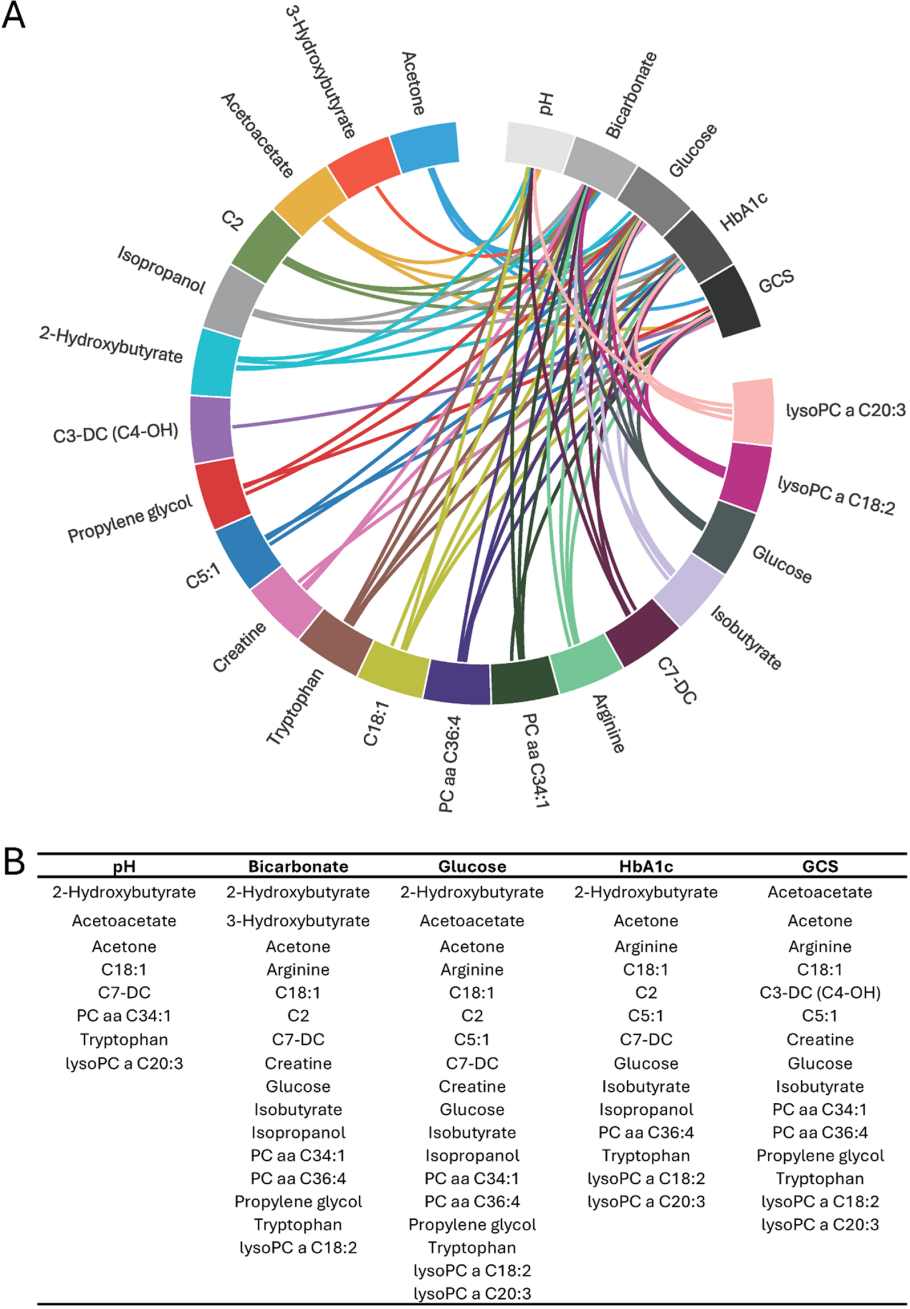
Metabolite panels associated with clinical variables. **A** Chord diagram illustrating the relationships between the top metabolites and clinical features. Clinical biochemistry and GCS were determined on admission to the pediatric critical care unit. Metabolites are positioned between 3:00 and 12:00, while clinical and demographic features are located between 12:00 and 3:00. Edges are color-coded by metabolite for enhanced clarity. **B** The edges highlight significant associations between metabolites and clinical features, as identified using the Lasso method, with details presented in tabular format

The clinical-metabolite associations are demonstrated graphically in Fig. 4. After conducting Lasso regressions, which perform linear regression with regularization to shrink the coefficients toward zero, we identified metabolite profiles that associated with clinically relevant characteristics of DKA. This analytic approach allowed us to determine which specific metabolites had non-zero coefficients, indicating their predictive value for clinical variables. We evaluated five clinical correlates: pH (RSS 0.08), bicarbonate (RSS 0.4), glucose (RSS 1.27), HbA1c (RSS 1.34), and GCS (RSS 0.67). Each clinical correlate was effectively associated by a panel of 8–18 metabolites.

### Pathways of interest

**Fig. 5:**
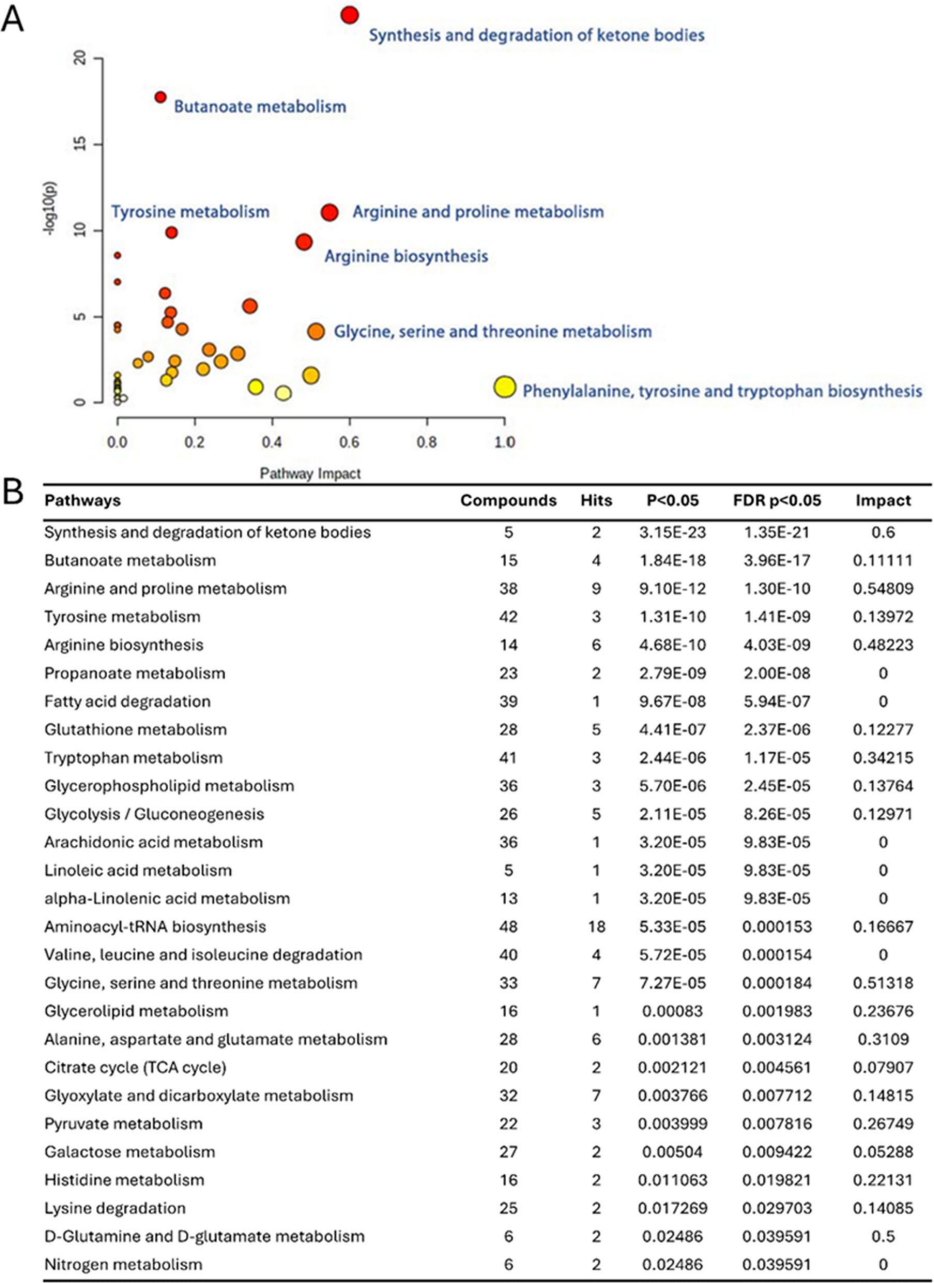
Functional Analysis showing enriched metabolic pathways as a list and graphically. **A** A scatter plot representing the results of a pathway enrichment analysis. The x-axis shows the pathway impact, while the y-axis represents the -log10(p) value, the statistical significance. Each dot represents a metabolic pathway, and the color of the dot corresponds to a different pathway category. The top five pathways, ordered by pathway effect scores or significance (p-value), are included with their names on the plot. **B** List of pathways enriched with the Globaltest method using the KEGG Homo Sapiens pathway library, comparing the control group with ongoing DKA. The statistical significance of each pathway is ranked, and the corresponding values are given. This list elucidates the differential metabolic pathway enrichment linked to DKA

Pathway enrichment analysis was performed using the KEGG Homo sapiens pathway library, and the results are displayed in Fig. 5, which includes both a list and a scatterplot of the most significant pathways. Figure 5A presents pathway impact versus statistical significance (− log10(p-value)), with a significance cutoff set at an adjusted p-value < 0.05. This analysis identified six prominently enriched pathways: “Synthesis and degradation of ketone bodies,” “Butanoate metabolism,” “Arginine and proline metabolism,” “Tyrosine metabolism,” “Arginine biosynthesis,” and “Glycine, serine, and threonine metabolism.” Notably, “Phenylalanine, tyrosine, and tryptophan biosynthesis” exhibited the highest impact, despite its lower statistical significance. The Globaltest method was used to determine significant pathways, focusing on pathway ranking rather than absolute p-values. Figure 5B provides a detailed list of pathways, including the total number of compounds in each pathway (Compounds), the number of compounds matching the user-uploaded data (Hits), the adjusted p-value < 0.05, the p-value adjusted for False Discovery Rate (FDR p < 0.05), and the pathway impact value derived from pathway topology analysis (Impact).

## Discussion

In this study, we used plasma metabolomics profiling with two complementary techniques (DI-LC/MS/MS and NMR), combined with multivariate statistics and machine learning, to identify distinct metabolite patterns in DKA patients compared to CON participants. We found 65 metabolites significantly altered in DKA, with 28 increased and 37 decreased. Feature ranking highlighted the key metabolites driving the differences between the cohorts. Pathway analysis revealed links between these metabolite changes and underlying pathophysiological processes, as well as clinical findings. Additionally, we developed metabolite panels associated with key clinical variables in DKA.

Elevated ketone bodies are a hallmark of DKA (Laffel 1999), and account for 14.9% of the variance between DKA and CON patients. Key ketone bodies identified include acetone, acetoacetate, and 3-hydroxybutyrate, all of which, along with their associated metabolic pathway, were significantly enriched. The most altered pathway in DKA was the “synthesis and degradation of ketone bodies,” consistent with the known pathophysiological mechanisms of metabolic acidosis in DKA (Dhatariya et al. 2020; Kraut and Madias 2010). Insulin deficiency, combined with elevated counter-regulatory hormones (Castellanos et al. 2020; Wolfsdorf et al. 2006), promotes the breakdown of non-esterified fatty acids and glycerol to generate Acetyl-CoA. While Acetyl-CoA normally enters the tricarboxylic acid cycle for ATP production, excess Acetyl-CoA is diverted to form 3-hydroxybutyrate and acetoacetate, with acetone produced via decarboxylation of acetoacetate (Dhatariya et al. 2020; Laffel 1999; Glaser 2005).

Further pathway analysis revealed enrichment in the metabolism of tyrosine and arginine, linking these amino acids to specific mechanisms in DKA (Hoffman et al. 2021; Szabó et al. 1991). Tyrosine plays a role in inflammation, immune-mediated pancreatic β-cell death, insulin resistance, and glucose metabolism, processes directly relevant to DKA. Tyrosine metabolism is well-established with new onset T1D but can persist beyond the initial disease onset. Inhibition of tyrosine-related proteins can improve insulin resistance (Marroqui et al. 2015; Gurzov et al. 2015; Stanford et al. 2017). Additionally, both the metabolism and synthesis of arginine were enriched. Arginine is essential for producing arginine-vasopressin, which may contribute to symptoms such as hypertension and hyperglycemia associated with glucagon production (Charlton et al. 1988; Sparapani et al. 2021; Henningsson and Lundquist 1998; Unger et al. 1970).

Acylcarnitines accounted for 23.4% of the variance between DKA and CON participants. These metabolites are essential for transporting long-chain fatty acids into the mitochondria, where they facilitate fatty acid β-oxidation (Dambrova et al. 2022; Reuter and Evans 2012). In DKA, insulin deficiency impairs fatty acid oxidation (Dhatariya et al. 2020; Calimag et al. 2023; Wolfsdorf et al. 2007), leading to elevated acylcarnitine levels, which likely reflect incomplete mitochondrial oxidation. Additionally, acylcarnitines can be derived from ketone bodies and amino acid degradation products (Dambrova et al. 2022; Lysiak et al. 1986; Simcox et al. 2017).

Phosphatidylcholines are the most abundant phospholipids in cell membranes and are vital for lipid metabolism, lipoprotein function, and cell signaling (Murphy et al. 1992; Chen et al. 2018). Our analysis found that phosphatidylcholines account for 9.7% of the variance between DKA and CON patients. Insulin typically promotes rapid phospholipase D-dependent hydrolysis of phosphatidylcholines, so insulin deficiency in DKA may disrupt lipid metabolism. Additionally, changes in lysophosphatidylcholine contribute to 5.4% of the variance between the cohorts. Lysophosphatidylcholine is produced by partial hydrolysis of phosphatidylcholines, which removes one fatty acid group, and notably, it negatively regulates insulin action (Motley et al. 2002).

Our findings also highlight alterations in lipid metabolism, particularly through the enrichment of pathways related to butanoate and proline. Butanoate has been linked to lipid and glucose metabolism and is recognized as a highly ketogenic component from a nutritional perspective (St-Pierre et al. 2017; Zhang et al. 2021). In contrast, proline and its metabolism are associated with lipid signaling, involving interactions between autophagy and oxidized low-density lipoproteins, as well as regular circadian rhythms (Phang et al. 2010; Gachon et al. 2011).

We identified eight metabolites (2-hydroxybutyrate, acetoacetate, acetone, C18:1, C7-DC, PCaaC34:1, tryptophan, and lyso PC a C20:3) associated with pH levels in DKA patients. Notably, several of these metabolites (2-hydroxybutyrate, acetone, C18:1, C7-DC, PCaaC34:1) were also linked to blood bicarbonate levels. The accumulation of ketone bodies in DKA increases hydrogen ion concentration, leading to anion gap acidosis. These excess hydrogen ions bind to bicarbonate, reducing its levels and contributing to the observed decrease in pH (Kraut and Madias 2010; Aduen et al. 1995). While the accumulation of fatty acids and phosphatidylcholines may influence plasma acidity, their effects are context-dependent and should be interpreted within the broader metabolic framework.

DKA is associated with elevated glucose and HbA1c levels, with HbA1c reflecting chronic hyperglycemia over the past 2–3 months (Eyth and Naik 2023). Our analysis identified several metabolites, including ketones, phospholipids, and amino, carboxylic, and fatty acids, that were linked to HbA1c levels. Reduced insulin sensitivity impairs glucose utilization and promotes ketogenesis (Laffel 1999; Wolfsdorf et al. 2007). Additionally, the upregulation of pathways involving glycogenic amino acids like glycine, serine, and threonine underscores the role of hyperglycemia in driving ketosis (Felig et al. 1970).

The level of consciousness, as measured by the GCS, was associated with a metabolite panel that included both glucose and ketones. Altered consciousness in DKA has been linked to the hyperosmolar environment caused by elevated glucose levels (Nevo-Shenker and Shalitin 2021). While the brain can utilize ketone bodies (acetoacetate and β-hydroxybutyrate) for energy (Jensen et al. 2020), studies have shown that direct infusion of ketones can decrease levels of consciousness (Svart et al. 2018). Therefore, high concentrations of glucose and ketones, along with severe acidosis, may contribute to the transient effects of DKA on consciousness. Other metabolites, including amino acids, fatty acids, phospholipids, and creatinine, were also associated with GCS, but their effects appear more indirect, primarily due to alterations in energy metabolism and acid–base balance.

The pathway most significantly altered in our DKA cohort was the “synthesis and degradation of ketone bodies,” aligning with both the identified metabolites and the established pathophysiological mechanisms of metabolic acidosis in DKA (Dhatariya et al. 2020; Laffel 1999; Kraut and Madias 2010; Glaser 2005). Clinical decompensation in DKA is often more closely linked to the degree of ketosis than to hyperglycemia. In addition to this primary pathway, we identified enrichment in five other major metabolic pathways, consistent with the proteolytic effects of hyperglycemia through glycogenolysis and gluconeogenesis. Notable upregulation was observed in the metabolism of butanoate, arginine, proline, tyrosine, glycine, serine, and threonine (Hoffman et al. 2021; Szabó et al. 1991). Butanoate plays a critical role in lipid and glucose metabolism and is a highly ketogenic component (St-Pierre et al. 2017; Zhang et al. 2021). Proline metabolism is particularly relevant due to its involvement in lipid signaling, autophagy, oxidized low-density lipoproteins, and circadian rhythms (Phang et al. 2010; Gachon et al. 2011) The tyrosine family is significant in DKA for its roles in inflammation, insulin resistance, and glucose metabolism; inhibiting these proteins can improve insulin sensitivity (Marroqui et al. 2015; Gurzov et al. 2015; Stanford et al. 2017). Finally, glycine, serine, and threonine, as glycogenic amino acids, contribute to the severe hyperglycemia characteristic of DKA (Felig et al. 1970).

We also found enrichment in the “arginine biosynthesis” pathway. Arginine is critical for producing arginine-vasopressin, which may contribute to hypertension in DKA due to overexpression of counter-regulatory hormones (Charlton et al. 1988; Sparapani et al. 2021). Additionally, arginine stimulates glucagon production, which exacerbates hyperglycemia and acidosis in DKA (Henningsson and Lundquist 1998; Unger et al. 1970).

Our study provides a comprehensive analysis of the metabolite profile in pediatric DKA, though several limitations should be considered. First, while we included a balanced but limited number of matched participants, our findings remain statistically significant even after correcting for multiple comparisons. These results are consistent with those of similar studies, despite the lack of a specific focus on pediatric DKA metabolomics (Jahoor et al. 2021; Jin and Ma 2021). Second, we focused only on severe DKA patients to examine metabolic changes, but future studies should include a broader spectrum of DKA severity to improve generalizability. Third, differences in T1D duration between cohorts, with some participants experiencing DKA as their first manifestation, may have influenced the results. Finally, while we identified differentially expressed metabolites in DKA plasma, the absence of longitudinal samples limits our ability to track changes in metabolite levels over time and in response to treatment.

Severe insulin deficiency, coupled with elevated counter-regulatory hormones, leads to DKA. Our study aimed to provide a deeper, more comprehensive understanding of the underlying metabolic alterations in DKA beyond the typical biochemical markers demonstrated previously and provide a greater understanding of the metabolic changes associated with the pathophysiology. We identified significant disruptions in lipid metabolism and mitochondrial function, uncovering key metabolites that differentiate DKA patients from controls. Additionally, we established metabolite panels that correlate with clinical variables in DKA. Overall, these findings underscore the potential of metabolomics profiling as a powerful tool for uncovering the metabolic changes underlying DKA and advancing its pathophysiological understanding.

## Supplementary Information

Supplementary material 1.

## Data Availability

The datasets generated and/or analysed during the current study are available from the corresponding author on reasonable request.

## Abbreviations

T1D: Type 1 diabetes
DKA: Diabetic ketoacidosis
CON: Control
NMR: Nuclear magnetic resonance
MS: Mass spectrometry
HbA1c: Hemoglobin A1C
GCS: Glasgow coma scale
RSS: Residual sum of squares
FDR: False discovery rates
