# Supplementary material for: Metabolomic signature of pediatric diabetic ketoacidosis: key metabolites, pathways, and panels linked to clinical variables
Source: Mol Med. 2024 Dec 20;30:250. doi: 10.1186/s10020-024-01046-9 (PMC11660668; doi:10.1186/s10020-024-01046-9)
Supplement: Supplementary file 1 — Supplementary material 1. [file 10020_2024_1046_MOESM1_ESM.docx]

**Supplementary Table 1. A metabolite list with statistically significant changes associated with DKA.**

| **Metabolite** | **Fold Change** | **P-Value** | **P-Value (adj.)** | **Direction** |
| --- | --- | --- | --- | --- |
| 1. 3-Hydroxybutyrate | 88.142 | 1.65E-33 | 3.55E-31 | up |
| 1. Acetoacetate | 46.508 | 7.10E-24 | 1.53E-21 | up |
| 1. Glutamine | -2.845 | 5.82E-19 | 1.25E-16 | down |
| 1. C2 | 7.93 | 9.82E-19 | 2.11E-16 | up |
| 1. Acetone | 6.604 | 1.48E-16 | 3.18E-14 | up |
| 1. C3-DC (C4-OH) | 22.268 | 2.15E-16 | 4.62E-14 | up |
| 1. Methanol | -3.638 | 1.67E-15 | 3.60E-13 | down |
| 1. Isopropanol | 6.289 | 5.64E-13 | 1.21E-10 | up |
| 1. Carnitine | -3.626 | 4.26E-12 | 9.16E-10 | down |
| 1. 2-Hydroxybutyrate | 3.972 | 9.22E-11 | 1.98E-08 | up |
| 1. Arginine | -2.305 | 1.63E-10 | 3.51E-08 | down |
| 1. Propylene glycol | 11.876 | 6.08E-10 | 1.31E-07 | up |
| 1. Tryptophan | -2.068 | 8.05E-10 | 1.73E-07 | down |
| 1. Succinate | -1.913 | 7.32E-09 | 1.57E-06 | down |
| 1. PC aa C36:4 | 1.979 | 1.14E-08 | 2.45E-06 | up |
| 1. Choline | -1.9 | 2.64E-08 | 5.68E-06 | down |
| 1. Phenylalanine | -1.91 | 2.86E-08 | 6.15E-06 | down |
| 1. C18:1 | 2.532 | 5.35E-08 | 1.15E-05 | up |
| 1. Lactate | -2.93 | 8.11E-08 | 1.74E-05 | down |
| 1. PC aa C34:1 | 1.539 | 1.16E-07 | 2.49E-05 | up |
| 1. trans-OH-Proline | -2.102 | 1.90E-07 | 4.09E-05 | down |
| 1. Lysine | -1.919 | 2.28E-07 | 4.89E-05 | down |
| 1. PC aa C38:4 | 1.535 | 3.49E-07 | 7.50E-05 | up |
| 1. Creatine | 2.602 | 4.16E-07 | 8.94E-05 | up |
| 1. lysoPC a C20:3 | -2.866 | 4.93E-07 | 0.000106 | down |
| 1. lysoPC a C18:2 | -2.188 | 6.07E-07 | 0.00013 | down |
| 1. Ornithine | -2.297 | 7.66E-07 | 0.000165 | down |
| 1. C10:2 | -1.963 | 1.54E-06 | 0.00033 | down |
| 1. Glycine | -2.542 | 2.42E-06 | 0.000519 | down |
| 1. PC ae C34:3 | -1.641 | 2.50E-06 | 0.000537 | down |
| 1. lysoPC a C18:0 | -2.021 | 2.72E-06 | 0.000586 | down |
| 1. Formate | -1.888 | 2.81E-06 | 0.000604 | down |
| 1. SM C16:1 | 1.39 | 2.90E-06 | 0.000623 | up |
| 1. PC aa C32:0 | 1.453 | 3.50E-06 | 0.000753 | up |
| 1. Methionine | -1.77 | 3.76E-06 | 0.000808 | down |
| 1. Valine | -2.168 | 5.86E-06 | 0.00126 | down |
| 1. Citrulline | -2.215 | 6.50E-06 | 0.0014 | down |
| 1. Serine | -1.784 | 7.50E-06 | 0.00161 | down |
| 1. PC aa C32:1 | 1.566 | 1.03E-05 | 0.00222 | up |
| 1. PC ae C36:1 | 1.33 | 1.06E-05 | 0.00229 | up |
| 1. C16 | 1.976 | 1.27E-05 | 0.00273 | up |
| 1. Proline | -2.12 | 1.30E-05 | 0.0028 | down |
| 1. PC aa C38:5 | 1.56 | 1.43E-05 | 0.00306 | up |
| 1. C12-DC | -1.518 | 1.66E-05 | 0.00356 | down |
| 1. C5:1 | 1.753 | 1.81E-05 | 0.0039 | up |
| 1. Threonine | -1.773 | 1.84E-05 | 0.00395 | down |
| 1. Ethanol | -2.287 | 1.97E-05 | 0.00424 | down |
| 1. PC ae C42:3 | -1.595 | 2.17E-05 | 0.00466 | down |
| 1. SM C18:0 | 1.455 | 2.18E-05 | 0.0047 | up |
| 1. C16:1 | 1.685 | 2.19E-05 | 0.0047 | up |
| 1. C14:1 | 2.261 | 2.90E-05 | 0.00624 | up |
| 1. Asparagine | -1.988 | 3.39E-05 | 0.00729 | down |
| 1. Creatinine | -2.098 | 3.63E-05 | 0.00779 | down |
| 1. Isobutyrate | 1.853 | 3.69E-05 | 0.00794 | up |
| 1. alpha-Aminoadipic acid | 2.74 | 3.94E-05 | 0.00847 | up |
| 1. PC aa C38:3 | -1.533 | 3.98E-05 | 0.00856 | down |
| 1. PC aa C38:6 | 1.75 | 6.97E-05 | 0.015 | up |
| 1. PC aa C42:4 | -2.103 | 7.04E-05 | 0.0151 | down |
| 1. PC aa C26:0 | 1.45 | 7.05E-05 | 0.0151 | up |
| 1. PC ae C44:3 | -2.826 | 7.90E-05 | 0.017 | down |
| 1. C14:2 | 1.97 | 8.81E-05 | 0.0189 | up |
| 1. Malonate | -1.927 | 0.000104 | 0.0223 | down |
| 1. Glutamic acid | -2.406 | 0.000182 | 0.0392 | down |
| 1. PC aa C36:2 | -1.63 | 0.00022 | 0.0474 | down |
| 1. Tyrosine | -1.452 | 0.000221 | 0.0476 | down |

**Supplementary Table 2. A list of the first 54 metabolites contributing to the variance between groups generated with a feature importance calculation using random ­­­forests (%).**

| **Metabolite** | **Importance** |
| --- | --- |
| 1. Acetone | 0.075 |
| 1. 3-Hydroxybutyrate | 0.070 |
| 1. Acetoacetate | 0.069 |
| 1. C2 | 0.057 |
| 1. Isopropanol | 0.057 |
| 1. 2-Hydroxybutyrate | 0.045 |
| 1. C3-DC (C4-OH) | 0.044 |
| 1. Propylene glycol | 0.037 |
| 1. C5:1 | 0.029 |
| 1. Creatine | 0.029 |
| 1. Tryptophan | 0.028 |
| 1. C18:1 | 0.026 |
| 1. PC aa C36:4 | 0.026 |
| 1. PC aa C34:1 | 0.022 |
| 1. Arginine | 0.021 |
| 1. C7-DC | 0.019 |
| 1. Isobutyrate | 0.019 |
| 1. Glucose | 0.019 |
| 1. lysoPC a C18:2 | 0.019 |
| 1. lysoPC a C20:3 | 0.019 |
| 1. C16 | 0.018 |
| 1. lysoPC a C18:0 | 0.016 |
| 1. Citrulline | 0.014 |
| 1. 3-Hydroxyisovalerate | 0.014 |
| 1. Ornithine | 0.013 |
| 1. C5 | 0.013 |
| 1. trans-OH-Proline | 0.012 |
| 1. PC ae C42:3 | 0.012 |
| 1. C0 | 0.011 |
| 1. Carnitine | 0.010 |
| 1. Glutamic acid | 0.010 |
| 1. PC aa C32:0 | 0.009 |
| 1. C16:1 | 0.008 |
| 1. PC aa C38:3 | 0.008 |
| 1. C14:1 | 0.008 |
| 1. C18 | 0.008 |
| 1. C14:2 | 0.007 |
| 1. Proline | 0.007 |
| 1. C16:2 | 0.006 |
| 1. C18:2 | 0.006 |
| 1. C12 | 0.006 |
| 1. PC ae C36:3 | 0.005 |
| 1. PC ae C34:3 | 0.005 |
| 1. PC aa C38:4 | 0.005 |
| 1. PC aa C40:1 | 0.005 |
| 1. lysoPC a C16:0 | 0.004 |
| 1. Succinate | 0.004 |
| 1. C16:1-OH | 0.004 |
| 1. PC aa C42:0 | 0.003 |
| 1. C14 | 0.003 |
| 1. PC ae C42:4 | 0.003 |
| 1. C14:1-OH | 0.003 |
| 1. PC aa C38:6 | 0.003 |
| 1. C18:1-OH | 0.002 |
